# **Accidental Adhesion of Both Hands with Super Glue: A Case Report**

**Published:** 2016-01

**Authors:** Veysel Murat Isik, Kadri Ozer, Melike Oruc, Koray Gursoy, Adile Turan, Ugur Kocer

**Affiliations:** Ankara Training and Research Hospital, Plastic, Reconstructive and Aesthetic Surgery Clinic, Ankara, Turkey

**DEAR EDITOR**

Cyanoacrylates (CAs) were first described in 1949 and their potential as adhesives were quickly recognised.^1^ Various homologues of CA adhesive have been studied and used, including methyl-, ethyl-, isobutyl-, isohexyl-, and octyl-CA.^1^ Commercial CA now has widespread use as an all purpose adhesive in various industries, however the main application of CA is as adhesives domestically. In the early 1960s, the formulation was changed to butyl cyanoacrylate for possible medical use, creating a less toxic product.^2^ Despite their increasing use in the household, especially medical staff has limited knowledge about the management of adverse effects of CA and that limited knowledge could be ended up with a catastrophe. T this report presents a case of CA glue adhesion and highlights the knowledge of the relevant emergent situation.

Our case was a 28-year-old, right handed man, working as a carpet upholsterer applied to the Emergency Department with adhesion of both hands to each other by super glue while he was covering the floor with carpet. At the time of contact with CA, to clean the glue, he rubbed his fingers to each other and then, washed his hands with water and soap. However, after a while he had a burning sensation in his hands and felt pain when he tried to detach his fingers. Patient’s both hands and fingers adhered to each other completely and almost symmetrically (Figure 1).

**Fig. 1:**
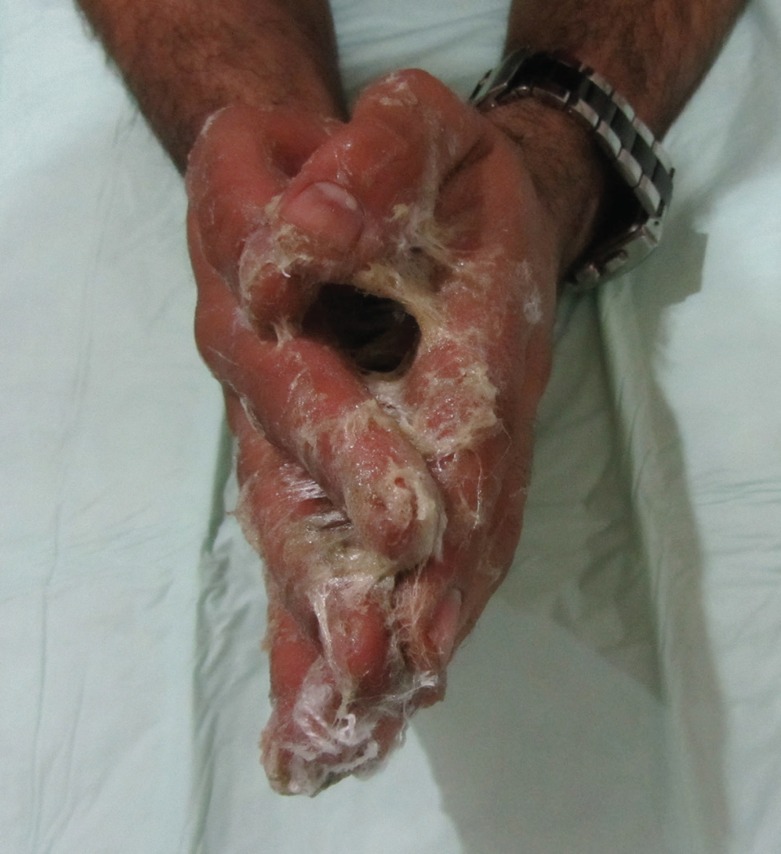
Adhesion of both hands and all fingers to each other resembling a prayer position

He applied to his occupational physican but he did not receive any treatment. He went back home and tried to solve glue by himself with a combination of some detergents, oil and warm water but could not succeed. A few hours later, the patient applied to the Emergency Department where the glue was tried to be solved by physical assistance and by liquid basic soap and alcohol. After the inefficient procedures applied by emergency staff, the patient was consulted to the Plastic Surgery Department for surgical operation.

The patient was noted to be fit and well, not suffering from any other medical problems nor taking any regular medications. However, he was anxious. On physical examination, patient’s hands looked like a prayer position. He stated that he couldvnot do any movement with his fingers. In treatment, acetone was applied to the patient’s hands and fingers and within a few seconds, the glue was dissolved (Figure 2) and the patient could use his hands and fingers freely again. 

**Fig. 2:**
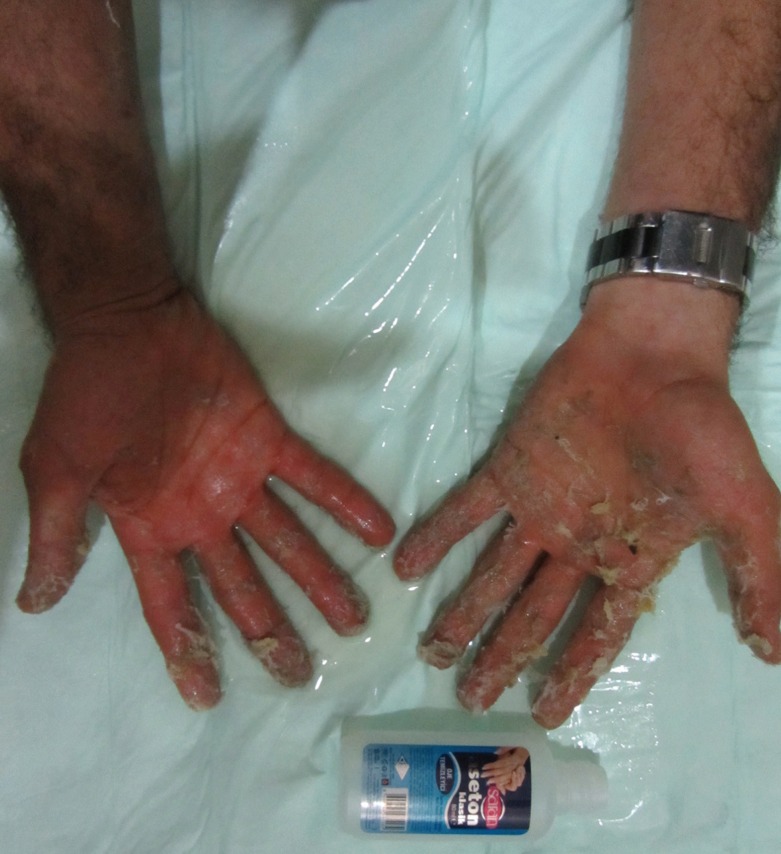
Glue which was dissolved by acetone within a few seconds

Cyanoacrylate (CA) cements contain a monomer group which is stabilized with a weak acid to prevent converting from liquid to solid in the container. To initiate the reaction, the acid stabilizer must be neutralized with a weak base. CA in the compound is converted from liquid to solid in the presence of weak bases such as water, alcohol, or blood. Airborne or surface absorbed water is usually sufficient to neutralize the weak acid.^3^ The amino acid content in skin, in addition to the water content, initiates the polymerization reaction very well. 

The CA molecules begin to link and the chains form a durable plastic mesh. The glue thickens and hardens until the movement of molecular chains ceases. Cyanoacrylate sets quickly, often in less than a minute and a normal bond reaches full strength in two hours and is waterproof.^4^

CAs have been available since the late 1950s^2^ and have multitude of uses, ranging from simple domestic applications, to those for industrial purposes.^4^ It has been said that 1-square-inch bond can hold more than a ton.^2^ Most of the commercially available adhesives are likely to be based on ethyl 2-CA and to a lesser extent on methyl 2-CA.^5^ Methyl 2-CA and ethyl 2-CA are clear, colourless liquids with strong, acrid odour.^5^ They easily enter into reaction with water to form a solid polymer. The substances are soluble or partially soluble in methyl ethyl ketone, toluene, N, N dimethylformamide, acetone, and nitromethane.^5^

The main disadvantage to cyanoacrylate use relates to its histotoxicity that may cause contact dermatitis and urticaria derived from the degradation of cyanoacrylate to formaldehyde and cyanoacetate compounds.^1^^,^^6^ CA glue can cause powerful, rapid exothermic reaction while curing and therefore, burn injury can occur in case of contact with skin especially when placed in contact with cotton and other fabrics.^4^ Full-thickness necrosis of thumb pad complicated by a secondary superinfection also has been reported before.^6^ The removal of hardened polymerized adhesive from the skin is likely to cause sloughing and irritation of the skin.

There are some basic steps to be taken when there is an exposure to the skin. First, soak the skin in warm soapy water to soften the glue as soon as possible.^4^ However, if there is too much hardened glue as it was in our case, this would not be very effective. Under these circumstances, use acetone nail polish remover which is a widely available solvent capable of softening cured CA by weakening its bonds. However, never use cotton swab as this can react violently with CA.^4^ Alternatively, pumice stone or nail emery board can also be used together with warm water to remove the glue.

CAs are commonly used as glue in households. Therefore, there is always the risk for spillage onto the skin particularly affects people do not use personal protective equipment. All health care providers have a possibility to meet such a patient in our report and so that, they should always remember the appropriate management of CA exposure to the skin and hand. Adhesion of the hand by CA glues can become more complicated by a health care provider who has limited knowledge about CA and wants to open the adhesion by excision while CA can be solved by a simple method using acetone. Our report focuses on the basic management of that widely used substance in case of skin and hand contact.

## CONFLICT OF INTEREST

The authors declare no conflict of interest.
